# A New Benzaldehyde Derivative Exhibits Antiaflatoxigenic Activity against *Aspergillus flavus*

**DOI:** 10.3390/jof9111103

**Published:** 2023-11-12

**Authors:** Usuma Jermnak, Paiboon Ngernmeesri, Chompoonek Yurayart, Amnart Poapolathep, Pareeya Udomkusonsri, Saranya Poapolathep, Napasorn Phaochoosak

**Affiliations:** 1 Department of Pharmacology, Faculty of Veterinary Medicine, Kasetsart University, Bangkok 10900, Thailand; fvetamp@ku.ac.th (A.P.); pareeya.s@ku.th (P.U.); fvetsys@ku.ac.th (S.P.); fvetanp@ku.ac.th (N.P.); 2 Department of Chemistry, Faculty of Science, Kasetsart University, Bangkok 10900, Thailand; paiboon.n@ku.th; 3 Department of Veterinary Microbiology and Immunology, Faculty of Veterinary Medicine, Kasetsart University, Bangkok 10900, Thailand; fvetcny@ku.ac.th

**Keywords:** antiaflatoxigenic activity, *Aspergillus flavus*, benzaldehyde derivative

## Abstract

Aflatoxin B1 (AFB1) is the most potent naturally occurring carcinogen for humans and animals produced by the common fungus *Aspergillus flavus* (*A. flavus*). Aflatoxin (AF) contamination in commodities is a global concern related to the safety of food and feed, and it also impacts the agricultural economy. In this study, we investigated the AFB1-inhibiting activity of a new benzaldehyde derivative, 2-[(2-methylpyridin-3-yl)oxy]benzaldehyde (MPOBA), on *A. flavus.* It was found that MPOBA inhibited the production of AFB1 by *A. flavus*, with an IC_50_ value of 0.55 mM. Moreover, the inhibition of conidiation was also observed at the same concentration. The addition of MPOBA resulted in decreased transcript levels of the *aflR* gene, which encodes a key regulatory protein for the biosynthesis of AF, and also decreased transcript levels of the global regulator genes *veA* and *laeA*. These results suggested that MPOBA has an effect on the regulatory mechanism of the development and differentiation of conidia, leading to the inhibition of AFB1 production. In addition, the cytotoxicity study showed that MPOBA had a very low cytotoxic effect on the Madin-Darby canine kidney (MDCK) cell line. Therefore, MPOBA may be a potential compound for developing practically effective agents to control AF contamination.

## 1. Introduction

Aflatoxins (AFs) are a group of naturally occurring and structurally related toxic secondary metabolites produced mainly by *A. flavus* and *A. parasiticus*. The major AFs are designated AFB1, AFB2, AFG1, and AFG2, which are of significance as direct contaminants of foods and feeds [1,2]. Among these, AFB1 is a great concern because it is a potent natural carcinogen. The International Agency for Research on Cancer [3] classified AFB1 as a Group 1 human carcinogen. As well as its adverse effects on the health of humans and animals, AFB1 also has a serious impact on the agricultural economy [4,5]. Numerous reports and studies have revealed that contamination of AFB1 in various agriculture commodities is increasing worldwide, particularly in tropical and subtropical countries [6,7,8]. AFB1 contamination remains a massive problem in developing countries in Southeast Asia, Africa, and the Middle East [9,10]. Moreover, it is impossible to eliminate AFB1 completely from food and feed, since it is heat-resistant and soluble in intermediate polar solvents [11]. Several decontamination and detoxication strategies for AFB1 have attempted to resolve the problem of AFB1 contamination [12,13,14]. So far, few practical methods to control AFB1 contamination in food and feed have been developed [15,16]. Therefore, it is critical to develop effective methods for preventing AFB1 contamination.

The biosynthesis of AF is regulated by the coordinated expression of a set of genes clustered within a 75 kb region of the fungal genome [17,18]. These genes encode for enzymes directly involved in the multistep biosynthetic pathway, including two regulatory genes (*aflS* and *aflR*) and structural genes such as *aflD* and *aflQ* [19]. Nevertheless, the biosynthesis of AF is also regulated by the genes encoding the global regulator of development and secondary metabolites such as *veA* and *laeA* [20,21]. It has been revealed that the accumulation of AF is orchestrated by a complex regulatory network and is activated by several environmental cues. Therefore, each of these genes might be a promising target for new effective method to control *A. flavus* and the production of AFB1. Currently, chemical-based control is still the most effective method for controlling post-harvest AF contamination. However, this is not practically useful because of the problems of food quality after treatment, toxicity, and residues in the food chain [22,23]. Therefore, exploring new effective and safe substances for preventing the production of AFs is desirable. Benzaldehyde is a plant metabolite and is similar to oxygenated terpenes. It is considered to be an environmentally safe compound because it is biodegradable [24]. Benzaldehyde is widely used as an antimicrobial compound [25] as well as a fungicide [26]. It is used in cosmetics as a denaturant and a flavoring agent, and is generally regarded as a safe food additive in the United States and European Union [27]. Furthermore, benzaldehyde has exhibited potent heat-sensitizing action to enhance the efficacy of sanitary measures against *A. flavus* contaminating crop seeds [28]. From this viewpoint, finding a new promising benzaldehyde derivative for controlling the contamination of foodstuffs and feeds by AFB1 and discovering its mechanism of action are required to provide a sustainable way to combat AF contamination.

**Scheme 1:**
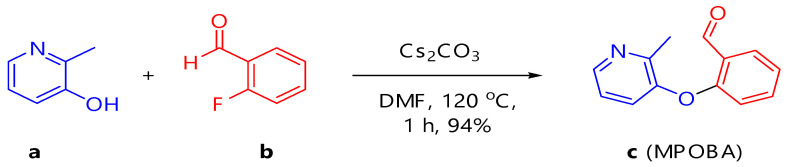
One-step synthesis of 2-((2-methylpyridin-3-yl)oxy)benzaldehyde (MPOBA) (**c**) from 3-hydroxy-2-methylpyridine (**a**) and 2-fluorobenzaldehyde (**b**).

In 2020, MPOBA was reported to possess anticancer activity against the human colorectal cancer cell line (HCT-116) with an IC_50_ value of 24.95 µM [29]. The synthesis of this compound was very simple and efficient. It was easily prepared in one step from the reaction of 3-hydroxy-2-methylpyridine (a) and 2-fluorobenzaldehyde (b) in the presence of an inexpensive reagent, cesium carbonate (Cs_2_CO_3_) in dimethylformamide (DMF) (Scheme 1). The yield of MPOBA (c) was also excellent (94%), and the reaction time was very short (1 h), making the compound ideal for large-scale production. Therefore, it would be worthwhile to further investigate the biological activities of MPOBA. The aim of this study was to investigate the in vitro antiaflatoxigenic activity of this new benzaldehyde derivative against *A. flavus* and elucidate its mechanism of action by determining its effects on fungal growth, the production of AFB1, the development of conidia, and the expression of related genes. In addition, a cytotoxicity study of MPOBA was also evaluated on a normal animal cell line. 

## 2. Materials and Methods

### 2.1. Fungal Strain and Culture Conditions

The *A. flavus* 3041 strain was obtained from the Postharvest Technology Research Group, Postharvest and Processing Research and Development Office, Department of Agriculture Bangkok, Thailand, and was used as a producer of AFB1. The strain was maintained on potato dextrose agar (PDA) (Difco, Sparks, MD, USA). A spore suspension was prepared from a two-week-old culture at a concentration of 10^6^ CFU/mL and was used as the inoculum.

### 2.2. Reagents and Chemicals

The synthesis and characterization of MPOBA was described in detail by Thongaram et al. [29]. The AFB1 standard, trifluoroacetic acid (TFA), and phosphate-buffered saline (PBS; pH 7.4) were purchased from Sigma-Aldrich (St. Louis, MO, USA). Deionized distilled water was prepared in the laboratory using the Milli-Q purification system (Millipore, Bedford, MA, USA). Acetonitrile (MeCN), methanol (MeOH), chloroform (CHCl_3_), trifluoroacetic acid (TFA), and formic acid (FA) of high-performance liquid chromatography (HPLC) grade were purchased from RCI Labscan (Bangkok, Thailand).

### 2.3. Antifungal and Antiaflatoxigenic Activities of MPOBA in a Liquid Medium

Potato dextrose broth (PDB) (Difco, Sparks, MD, USA) was added to a 24-well microplate at 1 mL per well. MPOBA was dissolved in methanol and added to the wells at final concentrations of 0, 0.35, 0.55, 0.75, 0.95, or 1.15 mM (the final concentration of methanol was 0.2% *v*/*v*). A spore suspension (30 µL) was inoculated into the medium and incubated statically for 3 days at 30 °C. Each treatment was replicated six times. After 3 days of incubation, the culture broth from each well was filtered through Miracloth to obtain the mycelia and the culture filtrate. The obtained mycelia were washed with 5 mL of distilled water and collected in a 1.5 mL microtube. After drying the mycelia at 80 °C for 3 h, the mycelial weight was calculated by subtracting the weight of a 1.5 mL microtube without mycelia from the total weight. For the extraction of total AFB1 content from 1 mL of the culture filtrate, AFB1 was extracted by vortex-assisted dispersive liquid–liquid microextraction (VADLLME) [30]. CHCl_3_ (400 μL) was added to 1600 μL of MeCN. The mixture was then injected into a 15 mL centrifuge tube that contained 1 mL of the culture filtrate. The tube was vigorously shaken by vortexing for 1 min. Then, the tube was centrifuged for 5 min at 3800 rpm. The lower organic phase was transferred to a new centrifuge tube and evaporated to dryness under a gentle stream of nitrogen at 45 °C. The dried extract was reconstituted in 900 μL of MeCN, followed by the addition of 100 μL of TFA, and then the mixture was incubated for 15 min at 15 °C. The derivatized solution was then vigorously shaken by vortexing for 1 min and filtered with a 0.22 μm PTFE membrane filter before ultra-high-performance liquid chromatography (UHPLC) analysis. The AFB1 content was determined by calculating the total amount present in the liquid medium.

### 2.4. UHPLC Analysis of AFB1

Samples were analyzed for AFB1 using an Agilent 1290 Infinity II high-speed UHPLC system (Agilent Technologies, Santa Clara, CA, USA) equipped with a fluorescence detector. Separation was performed by a Zorbax Eclipse XDB C18 system (2.1 × 50 mm, 1.8 µm column) with a temperature set to 40 °C. The samples were eluted for 10 min with an isocratic elution phase of 20% MeCN in water containing 0.1% FA. The flow rate was 0.3 mL/min, the injection volume was 5 µL, and the retention time of AFB1 was 3.9 min. The excitation and emission wavelengths were 360 and 430 nm, respectively. Linearity was assessed by constructing five-point calibration curves over the calibration range of 2.5–500 ng/mL for AFB1. The values of the limit of detection and the limit of quantification of AFB1 are 0.66 and 2.21 ng/mL, respectively.

### 2.5. Effect of MPOBA on the Expression of Genes Involved in AFB1 Biosynthesis

*A. flavus* 3041 was cultured for 3 days under the culture conditions described above with MPOBA at concentrations of 0, 0.55, 0.75, and 0.95 mM. The culture broth was filtered to obtain the mycelia. The total RNA was isolated using the GeneJet RNA Purification kit (Thermo Fisher Scientific, Waltham, MA, USA). A 500 mg sample of the mycelial cake was homogenized in liquid nitrogen. The homogenized samples were resuspended in 500 μL of a lysis solution/2-mercaptoethanol mixture provided by the kit. The samples were vigorously shaken for 30 s and incubated at 65 °C for 10 min. All further procedures were essentially the same as the manufacturer’s protocol. To remove genomic DNA contamination, the samples were treated with RQ1 RNase-Free DNase (Promega, Madison, WI, USA) according to the manufacturer’s instructions. The RNA’s concentration and purity (A260/A280 ratio) were spectrophotometrically determined using a NanoDrop™ One/OneC microvolume UV-vis spectrophotometer (Thermo Fisher Scientific, Waltham, MA, USA). First-strand cDNA was obtained using the SuperScript^®^ III First-Strand Synthesis kit for reverse transcription polymerase chain reaction (RT-PCR) (Thermo Fisher Scientific, Inc.) according to the manufacturer’s protocol. Briefly, oligo (dT) primers (50 µM), a dNTP mix (10 mM), and RNase-free water were added to a final volume of 10 µL. The protocol for RT-PCR was 65 °C for 5 min, 50 °C for 50 min, 85 °C for 5 min, and 37 °C for 20 min. Six important genes in the biosynthesis pathway of AFs were used, including *aflR*, *aflS*, *aflD*, *aflQ*, *laeA*, and *veA* [31,32,33]. *β-actin* was used as the endogenous control due to its relatively stable expression level. All primers used for quantitative real-time PCR (qRT-PCR) are shown in Table 1. The relative mRNA expression levels were calculated using the 2^–∆∆ Cq^ method. The transcriptional level of all genes was determined in five replicates by qRT-PCR using a CFX 96 Touch real-time PCR detection system (Bio-Rad, Hercules, CA, USA). The qRT-PCR was performed using a reaction mixture containing 2 μL of the cDNA template, 10 μL of the QuantiTect SYBR Green PCR kit (Qiagen, Hilden, Germany), and 1 μL of each primer. The qRT-PCR protocol was as follows: initial activation at 95 °C for 10 min; denaturation at 95 °C for 15 s, annealing at 60 °C for 30 s, and extension at 72 °C for 30 s (40 cycles). A melt curve was generated by heating the sample from 72 to 95 °C with continuous measurement of the fluorescence to quantify the PCR product.

### 2.6. Effect of MPOBA on Sporulation in A. flavus

To determine the effect of MPOBA on sporulation in *A. flavus*, a spore suspension of the conidia of *A. flavus* was inoculated into PDB medium with MPOBA at concentrations of 0, 0.55, 0.75, and 0.95 mM [34,35]. The plates were incubated at 30 °C for 14 days. The growth and sporulation of each tested group were qualitatively examined by morphological methods, including the appearance of the colony under a stereomicroscope (SZ2-ILST, Olympus, Japan) and the microscopic morphology under a light microscope (BX43, Olympus, Japan) on Day 3, Day 7, and Day 14 of incubation at 30 °C. Each tested group was repeated five times. Semi-quantification of sporulation in *A. flavus* was determined using a spectrophotometer on Day 14 of incubation. Briefly, each tested group’s hyphae and conidia cells were resuspended in 1 mL of a sterile saline solution. The plates were sealed with parafilm, vigorously shaken at 200 rpm for 15 min, and allowed to settle for 5 min. The upper homogeneous layer was transferred, topped up to 3 mL with a sterile saline solution, and measured at an optical density of 530 nm [36,37].

### 2.7. Cytotoxicity Assay Using (3-(4,5-Dimethylthiazol-2-yl)-2,5-Diphenyltetrazolium Bromide (MTT Assay)

The Madin-Darby canine kidney (MDCK) cell line was cultured in Dulbecco’s modified Eagle’s medium (DMEM) containing 4.5 g/L glucose and L-glutamine (Corning, Corning, NY, USA) with 10% fetal bovine serum, 100 units/mL of penicillin, 100 µg/mL of streptomycin, and 0.25 µg/mL of Gibco Amphotericin B (Gibco, Carlsbad, CA, USA). The cultures were incubated at 37 °C in 5% CO_2_ at 95% humidity. The cells were harvested after they reached 70–80% confluence, cultured in 0.25% trypsin-EDTA (Corning, NY, USA) and subcultured regularly.

The MTT assay is a colorimetric assay used to detect cell proliferation. The principle is that the NADH-dependent oxidoreductase enzyme, which is available in viable cells, reduces the yellow MTT formazan into insoluble purple formazan crystals. The crystals can be dissolved using the solubilizing agent. The darker the solution, the higher the number of viable cells. 

MDCK cells were cultured in 96-well plates at a density of 5000 cells/well in DMEM with 10% FBS for 24 h to reach 70% confluence. Then the cells were treated with appropriate concentrations of MPOBA and incubated for 24 h. Next, 10 µL of MTT (50 mg/mL; 3-(4,5-dimethylthiazol-2-yl)-2,5-diphenyltetrazolium bromide) was added to the culture medium, and the plates were incubated for a further 4 h according to the manufacturer’s protocol. Then 100 μL of the solubilization solution was added to each well to solubilize the water-insoluble purple formazan crystals, followed by overnight incubation at 37 °C in 5% CO_2_. Doxorubicin (2 µg/mL) and 0.1% dimethyl sulfoxide (DMSO) were used as the positive cytotoxic control and the negative control, respectively. The absorbance at 570 nm was measured using a Synergy H1 hybrid multi-mode microplate reader (BioTek, Winooski, VT, USA). The experiment was performed in four replicates. The absorbance values of the treated cells were expressed as the percentage of cytotoxicity relative to the untreated control cells.

### 2.8. Statistical Analysis

The data generated in this study are expressed as the mean ± SD of at least four independent experiments. Statistical differences between two groups were analyzed using a Mann–Whitney nonparametric test. For the cytotoxicity assay, statistical differences between groups were analyzed using Kruskal–Wallis followed by Dunn’s multiple comparisons post hoc tests. Statistical analysis was performed using GraphPad Prism 6.0 (GraphPad Software, Inc., La Jolla, CA, USA). Values with *p* < 0.05 were considered to indicate a statistically significant difference. The production of AFB1 and fungal growth were quantified as the percentage of inhibition in relation to the control treatment without MPOBA using the following equation.

Percentage inhibition of the AFB1 production or fungal growth

(%) = (C − T/C) × 100;

where C is the concentration of AFB1 produced (ng) or the mycelial dry weight (mg) of the control plates (without MPOBA), T is the concentration of AFB1 produced or the mycelial dry weight of the MPOBA-treated plates.

## 3. Results

### 3.1. Effects of MPOBA on the Production of AFB1 and the Fungal Growth of A. flavus

**Figure 1:**
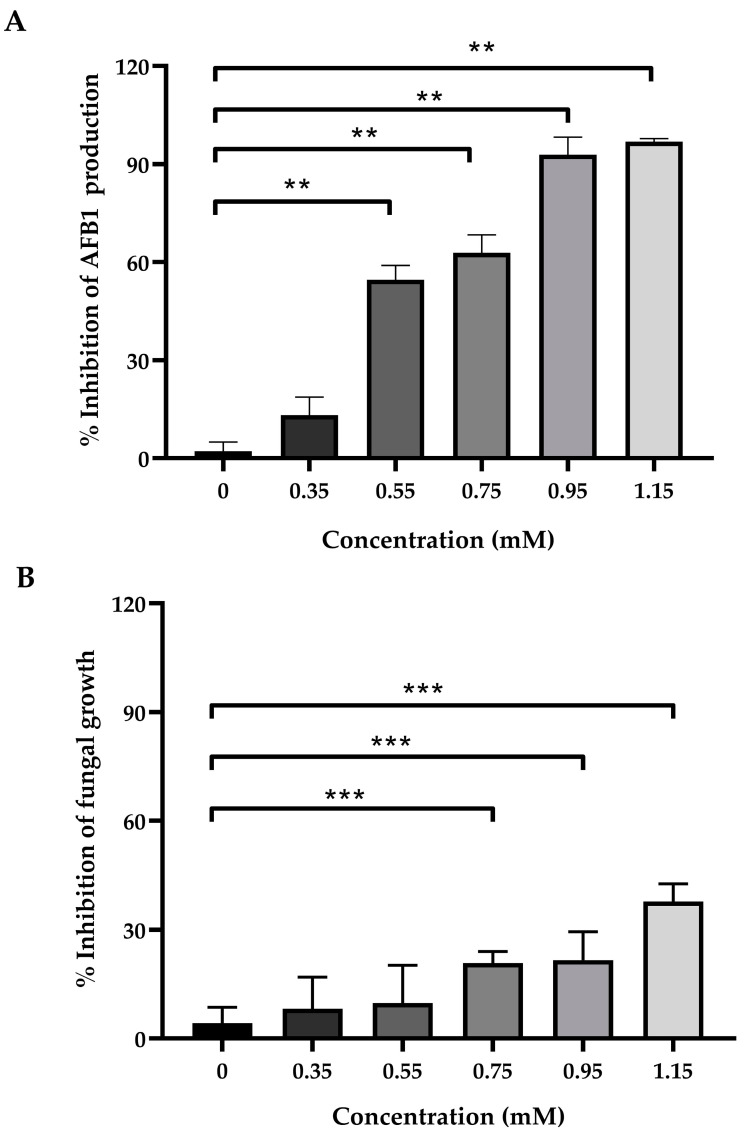
Effects of MPOBA on the production of AFB1 (**A**) and the fungal growth (**B**) of *A. flavus* 3041. Each bar shows the percentage of inhibition compared with the control. Each data point indicates the mean of six separate experiments ± SD. Statistical differences between the two groups were analyzed using the Mann–Whitney nonparametric test. Significant differences from the control are indicated as follows: ** *p* < 0.01 and *** *p* < 0.001.

The inhibitory activity of MPOBA on the production of AFB1 and the mycelial growth of *A. flavus* was examined at concentrations of 0, 0.35, 0.55, 0.75, 0.95, and 1.15 mM in a liquid medium. After 3 days of cultivation, the amount of AFB1 in the culture supernatant and the weight of fungal mycelia were measured (Figure 1A,B). The inhibitory effect of MPOBA was compared with the control (0 mM). The amount of AFB1 decreased in a concentration-dependent manner through the addition of MPOBA, with an IC_50_ value of 0.55 mM. The inhibitory activity of MPOBA against the production of AFB1 was almost 100%, while its fungal inhibitory activity was around 35% of the control at the highest concentration tested (1.15 mM). 

### 3.2. Effect of MPOBA on the Expression of Genes Involved in the Biosynthesis of AFB1

**Figure 2:**
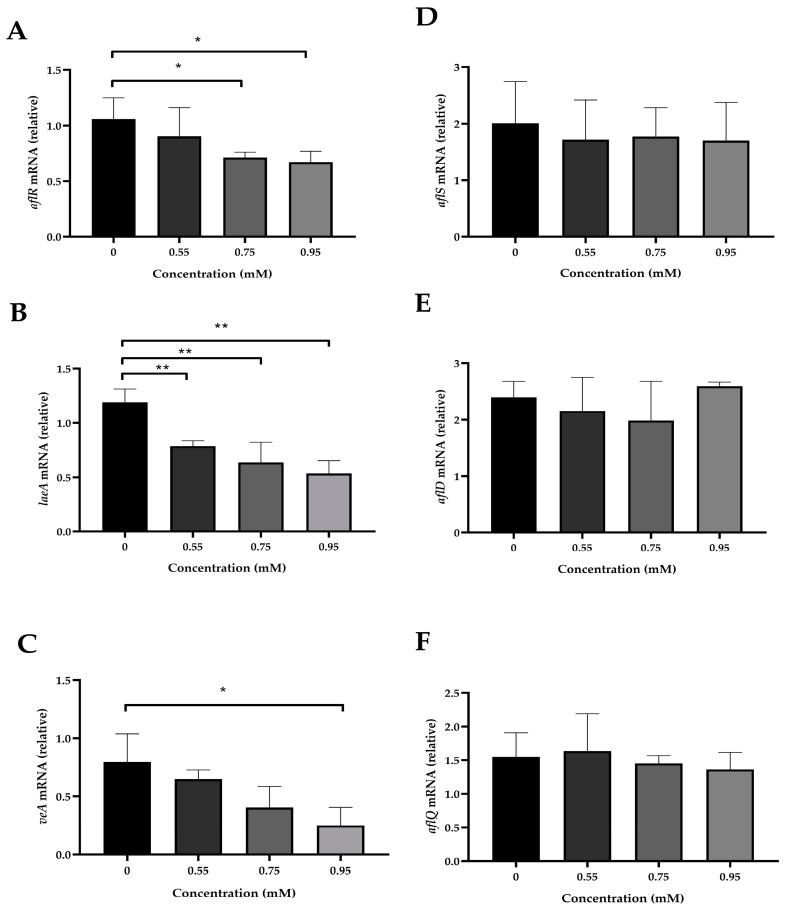
Effects of MPOBA on the expression of the *aflR*, *laeA*, *veA*, *aflS*, *aflD*, and *aflQ* genes involved in the biosynthesis of AFB1 (**A**–**F**). The relative abundance of mRNA was obtained by normalization to the expression of β-tubulin. Each data point indicates the mean of five separate experiments ± SD. Statistical differences between the two groups were analyzed using the Mann–Whitney nonparametric test. Significant differences from the control are indicated as follows: * *p* < 0.05 and ** *p* < 0.01.

To analyze the effect of MPOBA on the expression of genes involved in the biosynthesis of AFB1, *A. flavus* was cultured with various concentrations of MPOBA for 3 days. Six genes (*aflR*, *aflS*, *aflD*, *aflQ*, *laeA*, and *veA*) were investigated relative to the housekeeping gene (β-tubulin). The expression of *aflR* is known to positively regulate the transcription of genes encoding enzymes for the biosynthesis of AF. The *aflS* gene acts as a transcriptional enhancer to optimize the activity of AflR in the AF gene cluster [38]. The *aflD* gene is the key structural gene involved in the early stage of the AF biosynthetic pathway, while *aflQ* encodes an oxidoreductase which is required for the final steps involved in the conversion of sterigmatocystin to AFB1 [39]. The *laeA* and *veA* genes are global regulators that govern developmental and metabolic processes in many *Aspergillus* species. They are known to directly activate fungal growth and cellular development. In this study, when *A. flavus* was cultured with MPOBA, the transcription of the *aflR*, *laeA*, and *veA* genes was significantly suppressed in a dose-dependent manner (Figure 2A–C). However, the expression levels of *aflS*, *aflD*, and *aflQ* were not significantly impacted by MPOBA (Figure 2D–F). 

### 3.3. Effect of MPOBA on Sporulation in A. flavus

**Figure 3:**
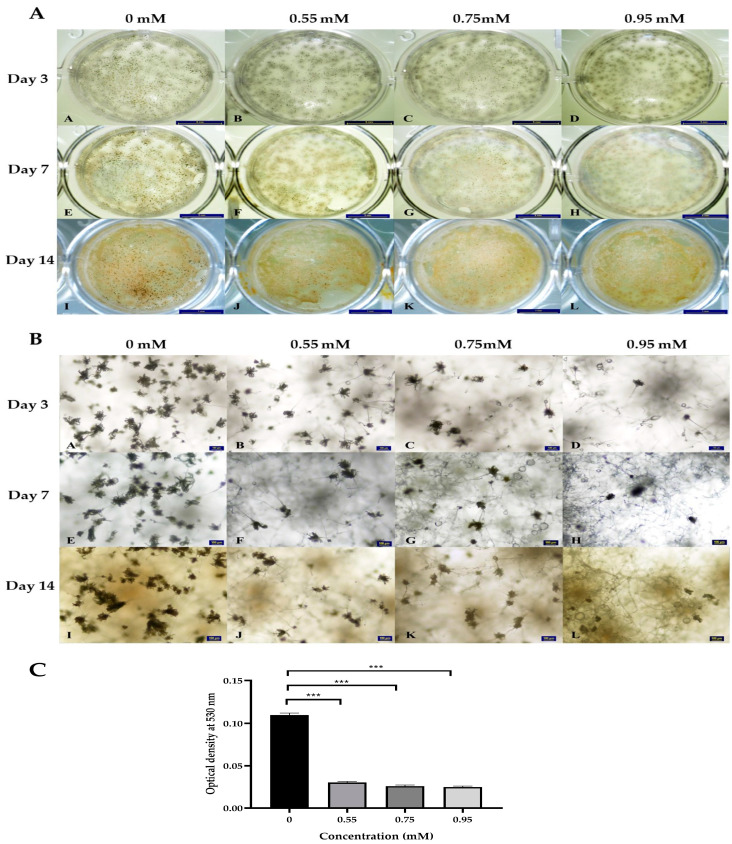
Effects of MPOBA on sporulation in *A. flavus*. Growth and sporulation were qualitatively examined by the appearance of the colony under a stereomicroscope (**A**) and the microscopic morphology under a light microscope (**B**) on Day 3, Day 7, and Day 14 of incubation. Semi-quantification of sporulation in A. flavus was determined using a spectrophotometer at an optical density of 530 nm on Day 14 of incubation (**C**). Each data point indicates the mean of five separate experiments ± SD. Statistical differences between the two groups were analyzed using the Mann–Whitney nonparametric test. Significant differences from the control are indicated as follows: *** *p* < 0.001.

When *A. flavus* 3041 was cultured in a liquid medium, MPOBA strongly affected the differentiation and morphology of the fungus. Colonies and the microscopic morphologies of all tested groups observed on Day 3, Day 7, and Day 14 of incubation at 30 °C are shown in Figure 3A and Figure 3B, respectively. Conidial heads of *A. flavus* appeared as green-yellowish points under a stereomicroscope. The aerial parts of *A. flavus*, including the conidiophores, vesicles, and conidial chains, were observed under a light microscope at 4×. Reduced sporulation was macroscopically and microscopically observed in line with the concentration of MPOBA compared with the control group throughout 14 days of incubation. Abundant conidial heads and chains were produced in the control group but dramatically decreased in the 0.55, 0.75, and 0.95 mM groups. In the semi-quantitative measurement of spore production regarding the OD 530 nm, the averages for the control, 0.55, 0.75, and 0.95 mM groups were 0.11, 0.03, 0.026, and 0.025, respectively (Figure 3C).

### 3.4. Effect of MPOBA on MDCK Cells

**Figure 4:**
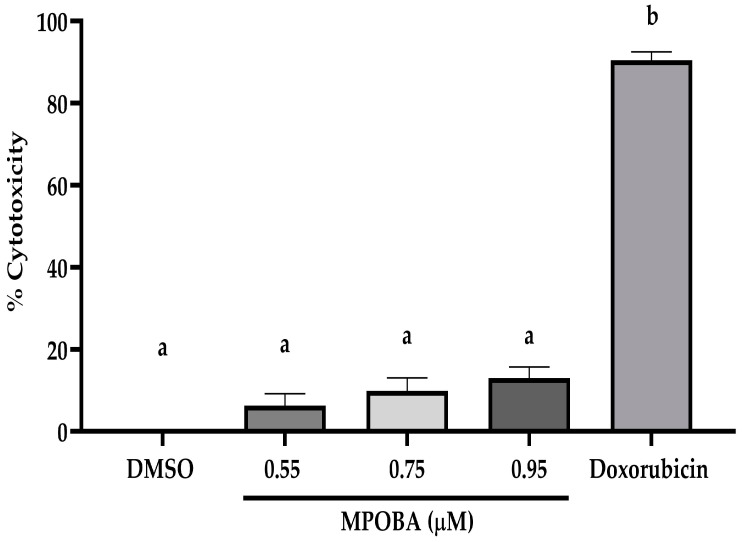
Cytotoxicity effect of MPOBA on MDCK cells. Cells were treated with MPOBA at various concentrations and cell viability was evaluated by MTT assays at 24 h. Doxorubicin (2 µg/mL) and 0.1% DMSO were used as the positive cytotoxic control and the negative control, respectively. Each data point indicates the mean of four separate experiments ± SD. Statistical differences between groups were analyzed using Kruskal–Wallis followed by Dunn’s multiple comparisons post hoc tests. Means with the same superscript are not significantly different (*p* < 0.05), whereas means with different superscripts are significantly different (*p* < 0.05).

A cytotoxicity study of MPOBA was carried out on a normal animal reference cell line (MDCK cells) using an MTT assay. This preliminary experiment showed that MPOBA has a low cytotoxic effect on MDCK cells at the highest concentration tested (12.97%), while doxorubicin was much more toxic to normal cells, killing almost all of them (90.45%) (Figure 4). 

## 4. Discussion

Several approaches have been proposed to limit contamination by AFB1 in crops [40]. One of them is to protect the food materials with safe chemical compounds. These compounds include the active constituents of herbs, essential oils, pesticides, microbial metabolites, and newly synthesized derivatives as inhibitors [41,42,43,44]. Therefore, the search for new compounds that are effectively antiaflatoxigenic, biodegradable, and toxicologically safe has motivated many studies. Currently, benzaldehyde is considered safe enough to be widely used in the food, beverage, and fragrance industries. Several studies have reported that benzaldehydes are generally regarded as safe (GRAS) food additives and flavoring substances in the United States and Europe. Various studies have suggested that benzaldehyde has extensive biological activities such as antimicrobial, insecticidal, antioxidant, anti-inflammatory, and antifungal activities [45]. A few reports are available showing the inhibition of fungal growth and the production of AF by some potent derivatives of benzaldehyde [46,47]. 

In *A. flavus*, the biosynthesis of AF is a multi-step process that involves at least 27 enzymatic reactions in the metabolic pathways. Gene clusters for the biosynthesis of AF are coordinately regulated, and their expression is coordinated by two cluster-specific transcriptional regulators required for the efficient production of AF (*aflR* and *aflS*) [48]. The *aflR* gene encodes the AflR transcription factor, which is a protein containing a zinc-finger DNA-binding motif. The AflR protein binds to at least 17 genes in the AF-biosynthetic cluster, resulting in the activation of the enzyme cascade and leading to the biosynthesis of AF [18]. The *aflS* gene is adjacent to the *aflR* gene and interacts with *aflR* to play a role in regulating the biosynthesis of AF [49]. The *aflD* gene encodes the ketoreductase needed for the conversion of the 1**′**-keto group in norsolorinic acid, the first stable precursor of AF, to the 1′-hydroxyl group of averantin [50]. The *aflQ* gene encodes an oxidoreductase that is involved in the formation of the AFB1 precursor hydroxylmethylsterigmatocystin, and it plays a role in the later stages of biosynthesis [51]. Previous studies have revealed that the disruption of the *veA* gene results in decreased formation of conidia, development of sclerotia, and formation of AF in *A. flavus* [52,53]. In addition, VeA also has a critical role in survival under oxidative stress in *A. flavus*. The disruption of *veA* has been shown to result in decreased oxidative stress tolerance in *A. flavus* and has led to complete inhibition of growth and the production of AFB1 [54]. Furthermore, several studies also identified and elucidated that LaeA, a nuclear protein, contains an *S*-adenosylmethionine binding motif which is required to activate the transcription of the gene cluster of AF biosynthesis in *A. flavus* [55]. A significant decrease in the production of AF was reported after the disruption of the *laeA* gene in *Aspergillus* strains [56]. 

**Figure 5:**
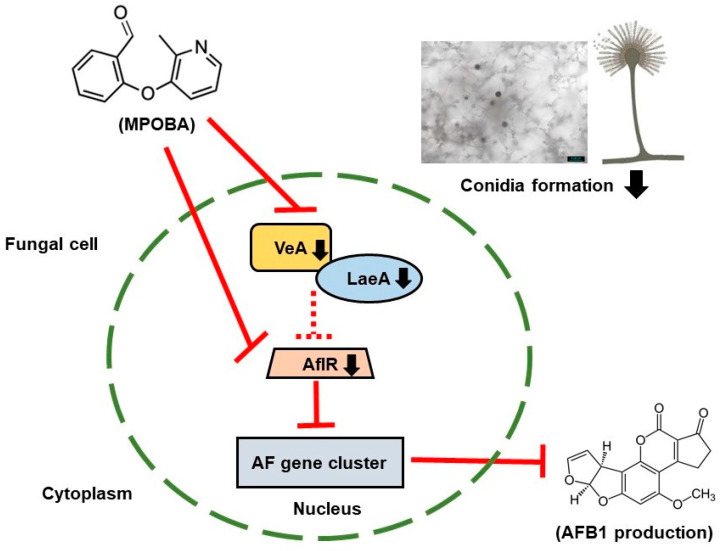
A possible AF-inhibiting mechanism of MPOBA in *A. flavus*.

In our current preliminary study, the new benzaldehyde derivative, MPOBA, was found to inhibit the production of AFB1 by *A. flavus* in a dose-dependent manner. When *A. flavus* was cultured in a liquid medium, the control culture without MPOBA germinated green conidia after 14 days of cultivation while inhibited formation of conidia was observed in the culture with MPOBA. This benzaldehyde derivative reduced the dry weight of *A. flavus* by 35% compared with the control. The addition of MPOBA led to a significant decrease in the expression of *aflR*. When *aflR* was suppressed, it resulted in lower transcript accumulation and decreased production of AF. In contrast, the expression of *afls* was not significantly decreased in the treatment groups, although a tendency of downregulation was observed. One key global regulatory complex of the biosynthesis of AF is the velvet complex, which consists of the proteins VeA, LaeA, and other regulators. Central to this complex is the cross-talk between LaeA, which is key to the epigenetic regulation of the biosynthetic pathway of AF [57], and VeA, a light-responsive regulatory protein that migrates from the cytoplasm to the nucleus in the absence of light to cooperate in the VeA complex [58]. In this study, the greatest effect of MPOBA was observed on *laeA* and *veA*, the global regulator genes belonging to the velvet complex. The expression levels of both genes were significantly downregulated in the treatment groups. Because MPOBA reduced the mRNA levels of *aflR*, *laeA*, and *veA*, it may affect the regulatory system that controls both the production of AFB1 and conidiation. The regulatory network of the biosynthetic pathway of AF is requisite to the global network of secondary metabolism in *A. flavus* [59]. Two possible AF inhibitory mechanisms of MPOBA in *A. flavus* are (1) through inhibiting the formation of the velvet complex, a protein complex comprising the LaeA and VeA proteins that regulate fungal secondary metabolism and the development of conidia, and (2) through the disruption of AflR, the regulatory protein that is critical for initiating the biosynthesis of AF. Moreover, the increase in the antiaflatoxigenic activity of MPOBA was associated with an inhibitory effect on the production of conidia (Figure 5). 

Several researchers have studied the antimicrobial, antifungal, and antiaflatoxigenic properties of aromatic aldehydes, including cinnamaldehyde (CA), cuminaldehyde (CU), lauraldehyde (LA), anisaldehyde, and other aldehydes [60,61]. Cinnamaldehyde, a major component of Chinese cinnamon oil, is recognized as one of the most effective plant extracts with antifungal and antiaflatoxigenic properties [62]. Wang et al. reported that the antiaflatoxigenic molecular mechanisms of CA in *A. flavus* involved (1) the reduction of fatty acid oxidation levels through the modulation of several oxidation-related genes. Consequently, this results in a significant decrease in the AF precursor acetyl-CoA, (2) the disruption of the redox system, subsequently activating antioxidant enzymes which are considered key elements in the regulation of AF-related genes, and (3) the disruption of fungal cell wall biosynthesis, ergosterol biosynthesis, and ATPase [63]. CA was also reported to inhibit spore germination by CA, leading to the suppression of mycelial development in *A. flavus*. Its antifungal effects were achieved by inducing alterations in cell morphology, causing damage to the cell membrane, and disrupting mitochondrial function through the interaction between calcium ions and reactive oxygen species. This ultimately leads to apoptosis of *A. flavus* [64]. In our present study, the almost complete inhibition of AFB1 production of MPOBA in *A. flavus* was observed at a concentration of 1.15 mM, while its fungal inhibitory activity was around 35%. These results are reasonable to that of Wang et al., who reported that the complete inhibition of AFB1 production of CU occurred at a concentration of 0.8 mM [63]. Additionally, Sun et al. reported that fungal growth and AF production were significantly inhibited by CU in a dose-dependent manner [65]. Furthermore, Xu, et al. also reported that CU exhibited direct antiaflatoxigenic activity by downregulating the gene expression related to AFB1 biosynthesis, including *aflR*, *aflS*, *laeA*, *veA*, and *velB*, and its antifungal activity was attributed to the disruption of the integrity of the membrane and the induction of necrosis of *A. flavus* [66]. The results of this study are in agreement with the findings of our study, suggesting that the suppressed production of AFB1 may be linked to its inhibitory effect on sporulation. In a study by Xie et al., LA in combination with geraniol, an acyclic monoterpene alcohol found in essential oils, could inhibit the germination of spores and the biosynthesis of AF in *A. flavus* [67]. Overall, several benzaldehydes have been identified to have potent antiaflatoxigenic activities related to their antifungal activities in *Aspergillus* species, in which their antifungal activities are through the disruption of the cellular antioxidation of fungi [68]. Possibly, the inhibited production of AFB1 of MPOBA may be related to its oxidative stress properties. However, the detailed regulatory mechanism and its targets still require further investigation. Furthermore, additional studies are required to explore the combined application of MPOBA and related compounds for their enhanced potential in combating AF contamination.

Generally, benzaldehyde is considered safe to be used in the food industry. Studies have reported very low acute toxicity or the absence of adverse effects when using benzaldehyde [69]. The cytotoxicity of MPOBA was examined in our study, and the results showed that MPOBA has a very low cytotoxic effect on canine kidney cells at the highest concentration tested. These efficacy and cytotoxicity studies of MPOBA suggest that this benzaldehyde derivative has the potential to control *A. flavus* and its production of AFB1. However, it is necessary to conduct further studies on the short- and long-term toxicity of MPOBA for practical applications in food and feed. Toxicological information is crucial to ensure the safety and suitability of MPOBA for human and animal health. Therefore, further investigations of the cytotoxicity on different normal cell lines as well as toxicity studies in animal models are still needed. 

## 5. Conclusions

To the best of our knowledge, this is the first report to evaluate the in vitro inhibitory activity of the new benzaldehyde derivative, MPOBA, against the production of AFB1 in *A. flavus.* The results showed that this compound significantly inhibited the production of AFB1 and the development of conidia of *A. flavus* in a dose-dependent manner. The effect of MPOBA mediated the downregulation of the *aflR*, *veA*, and *laeA* genes, which suggests that the mechanisms of MPOBA in *A. flavus* act through inhibiting the formation of the velvet complex and the disruption of *aflR*, which is critical for initiating the biosynthesis of AF. These preliminary results revealed that MPOBA may be a good compound for developing practically effective antifungal agents to control the production of AFB1 in grain. However, a deeper understanding of MPOBA’s mechanism of action for AFB1 and fungal growth inhibition, the molecular mechanism associated with oxidative stress response, and environmental factors need to be investigated. In addition, the appropriate application of MPOBA for controlling fungal growth and the production of AFB1 during the storage of grains, as well as the safety issues for human and animal health, should be investigated further. 

## Figures and Tables

**Table 1:** Primer sequences used for quantitative real-time PCR (qRT-PCR).

Gene	Primer (5′ to 3′)	PCR Product Size (bp)
*β-tubulin*	F: TCTTCATGGTTGGCTTCGCT R: CTTGGGGTCGAACATCTGCT	98
*aflR*	F: GATCTGGCTGGTCAGGAGCA R: CGCCTGAAACGGTGGTAGTG	204
*aflS*	F: TGGTGCGACCATATTTACA R: GGTTGGGTCACGAACTGTTT	94
*aflD*	F: ATGCTCCCGTCCTACTGTTT R: ATGTTGGTGATGGTGCTGAT	106
*aflQ*	F: TTAAGGCAGCGGAATACAAG R: GACGCCCAAAGCCGAACACAAA	599
*LaeA*	F: AAAGGTTGCTCGCTGGTACA R: GACTTCTGACGAAATGCGCC	121
*VeA*	F: TTGTCGTGTGCGGATTCG R: CTCATCGTAGTCGTATCATCG	79

## Data Availability

Data are contained within the article.
